# The Use of Oxandrolone in the Management of Severe Burns: A Multi-service Survey of Burns Centres and Units Across the United Kingdom

**DOI:** 10.7759/cureus.57167

**Published:** 2024-03-29

**Authors:** Jacob R Feathers, George Richardson, Alice Cornier, Nick Rebuffa, Brendan Sloan, Preetha Muthayya

**Affiliations:** 1 Trauma and Orthopaedics, Cwm Taf Morgannwg University Health Board, Llantrisant, GBR; 2 Trauma and Orthopaedics, Barts Health National Health Service Trust, London, GBR; 3 Medicine, Cwm Taf Morgannwg University Health Board, Llantrisant, GBR; 4 Radiology, Leeds Teaching Hospitals National Health Service Trust, Leeds, GBR; 5 Burns Department, Mid Yorkshire Teaching National Health Service Trust, Wakefield, GBR

**Keywords:** steroids, critical care, plastics, oxandrolone, burns

## Abstract

Introduction

Severe thermal burns are a catastrophic injury. Those surviving the initial insult are subject to life-long disability, prolonged hospital admission, nutritional issues and poor wound healing. Oxandrolone has been shown to reduce hospital duration and promote lean body mass. Despite not being licenced for use in burns trauma within the United Kingdom (UK), services across the country utilise Oxandrolone in the management of severe burns. We aim to analyse the use of Oxandrolone in major burns across burns services within the UK.

Methods

We conducted a survey across all burn centres and units across the UK. Any burns service provider with experience in patient management of patients sustaining burns with a total body surface area >15% was included. All services were identified using the British Burns Association website. We conducted a survey of all centres and units and contacted them via telephone through the trust’s switchboard. Responses were accepted from any healthcare staff familiar with the day-to-day in-patient care of patients on the ward. Services with no in-patient services were excluded.

Results

A total of 24 burns centres and services responded to our survey. Twelve of the respondents were in a burns unit and 12 were in a burns centre. Eight respondents were paediatric facilities, and the remaining 16 dealt with adult burns. In total, 16/24 (66.6%) services reported using Oxandrolone. Conversely, 8/24 (33.3%) burns services denied using Oxandrolone. 7/12 (58.3%) burns units use Oxandrolone in the management of burns. 5/12 (42.7%) burns units do not use Oxandrolone in severe burns. 9/12 (75%) of burns centres described using Oxandrolone, whilst the remaining 3/12 (25%) did not.

Discussion

Oxandrolone is used varyingly across burns services across the UK. Burns centres were more likely to use Oxandrolone compared to units. We also find that more paediatric services used Oxandrolone in comparison to adult services. Studies have shown that the benefit of Oxandrolone is not age-dependent. Further work is required to assess the impact of this medication on patients with severe burns and national guidance would help further improve burns management across the UK.

## Introduction

Severe thermal burns are devastating injuries, requiring immediate specialised care. They are defined as burns with >15% total body surface area (TBSA) [1]. Following insult, there is a profound and prolonged pathophysiological process where systemic inflammatory and compensatory anti-inflammatory processes are induced [2]. This leads to a myriad of complications, including hypovolaemic shock, immunosuppression and hypermetabolism [3]. As a result of this, and despite advancements in trauma medicine, most severe burns mortalities occur acutely following injury. Patients who survive the acute phase are subject to life-long disability; poor wound healing and nutritional issues [4]. 

Following major burns, the body is induced into a profound hypermetabolic state. This leads to rapid catabolism of protein reserves and is subsequently associated with reduced body mass, poor wound healing and sepsis [5]. This process prolongs patient admissions and is associated with increased morbidity and mortality [6].

Synthetic steroids have become an integral pharmacological therapy in the treatment of inflammatory and metabolic conditions. Oxandrolone is a synthetic testosterone analogue with six times the amount of anabolic activity compared to testosterone [7]. It is currently licenced to be used in various pathologies to maintain body mass in conditions such as Turner syndrome, acquired immune deficiency syndrome (AIDS) and alcoholic hepatitis [8]. A randomised control trial shows that Oxandrolone reduces the duration of hospital admission in children with severe burns [9]. Oxandrolone mitigates the catabolic pathophysiological processes that occur following a severe burn by improving lean mass and nutrition. This has subsequently been shown to improve wound healing, reduce the duration of the in-patient stay and improve survival [10,11].

Burns services within the United Kingdom (UK) follow a three-tier system of centres, units and facilities, with the most critically ill-treated at centres [12]. Oxandrolone is not currently licenced for the management of severe burns in the UK. Despite this, various burns services do use the drug in the management of acute burns patients. This often requires senior input and a multi-disciplinary approach in deciding to prescribe this medication. Our study aims to review the use of Oxandrolone across burns services within the UK. Despite current evidence, the role of Oxandrolone in major burns remains unclear. Our study is the first multi-service survey analysing the use of Oxandrolone in major burns.

## Materials and methods

Study parameters

We conducted a survey across all burn centres and units across the UK. Centres and units were identified using the British Burns Association website [12]. If the type of service was ambiguous, the National Health Service (NHS) website of the hospital was looked at to find out what level of burns provision the trust provides. Due to the focus of the study being on major burns, only burns centres and units were included. Burns facilities were not included within the study. 

In total, 44 burns services were identified across the United Kingdom using the British Burns Association website. Seven were subsequently excluded as they were classified as a burns facility. This left 35 burns centres and units. We then contacted each department via their trust's switchboard. A total of three attempts were made to discuss it with a member of staff within the department. If we were unsuccessful after three attempts, we documented the trust as a non-responder. 

Inclusion criteria

All burns centres and units as listed by the British Burns Association were included and subsequently contacted within our study. Responses were accepted from any healthcare staff or senior doctor familiar with the day-to-day in-patient care of patients within the department. 

Exclusion criteria

Burns facilities were not included during our study. This decision was made as burns facilities do not frequently deal with in-patient major burns patients and thus would not be appropriate for a survey. Trusts that do not provide an in-patient facility for the care of burns patients. Irish services were not included in the study.

Data collection and questionnaire

Our data was collected between November 2022 and December 2022 by authors NR, JF and AC. A questionnaire was developed and consisted of four questions. The questionnaire is shown in the Appendix 1 (Figure 6). On phoning the burns service, we requested to speak to a senior clinician or health care staff within the department. Prior to data collection, the respondent was informed that we were conducting a national survey of all units and centres across the UK in order to research the use of Oxandrolone within the UK burns practice. Our first question asked the respondent about their specific role within the department. We then enquired as to the level of burns service provided by the trust. If the service was a burns unit or centre, we then asked what demographic of patients they treat, either paediatric or adults. Our final question enquired as to whether Oxandrolone is used in the management of acute burns within their department. If the respondent was unsure, we then requested to speak to another member of staff within the department and re-started the questionnaire. We only accepted respondents who were senior doctors (senior trainee three or above) or health care staff who were familiar with the in-patient burns services. This included senior nurses, registered nurses, and pharmacists. We attempted to contact the service a maximum of three times.

## Results

**Figure 1:**
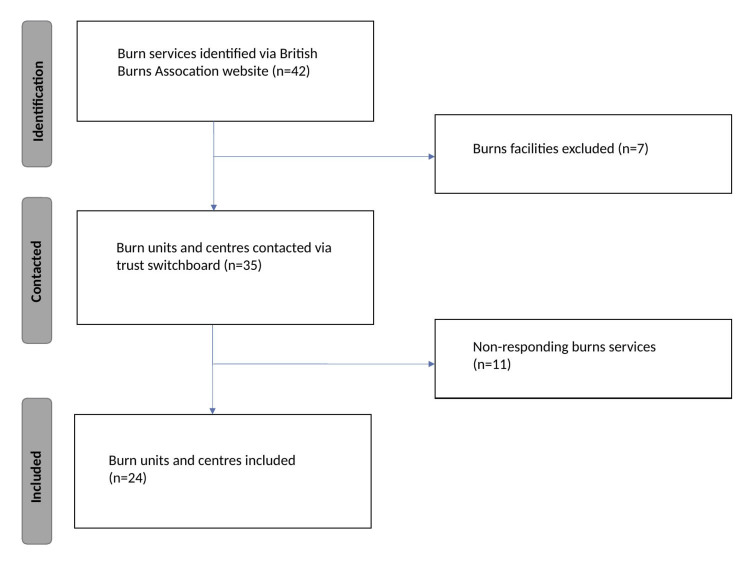
Flowchart detailing the process of data collection within the study. Forty-two burns services were identified from the British Burns Association website. Seven burns facilities were excluded as these services do not deal with acute major in patient burns. Thirty-five burns centres and units were called up to three times. A total of 24 burns units and centres responded across the United Kingdom.

There were 42 burns services identified across the UK. Seven burns facilities were excluded. A total of 35 burns centres and units were contacted. Eleven hospitals did not respond or did not meet inclusion/exclusion criteria after three attempts. There were subsequently 24 burns units and centres included in the the analysis of the results within our study. This is shown in Figure 1.

**Figure 2:**
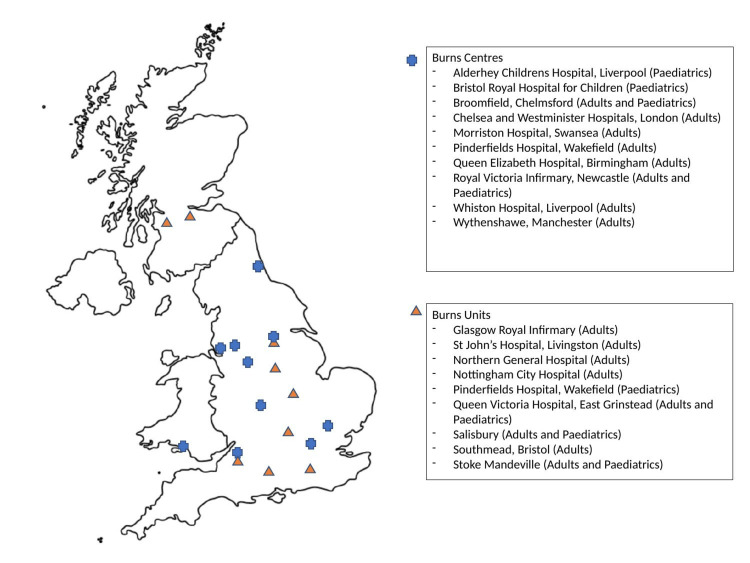
Map of the UK with the included burns services responding to our survey.

Across the UK, 12 burns centres responded to our survey. This includes four paediatric burn centres and eight adult burns centres. There were also 12 responses from burns units, comprising eight adult and four paediatric burns unit facilities. These services were spread across the UK and included England, Wales and Scotland. The distribution of responding services is shown in Figure 2, along with the type of service and patient category.

**Figure 3:**
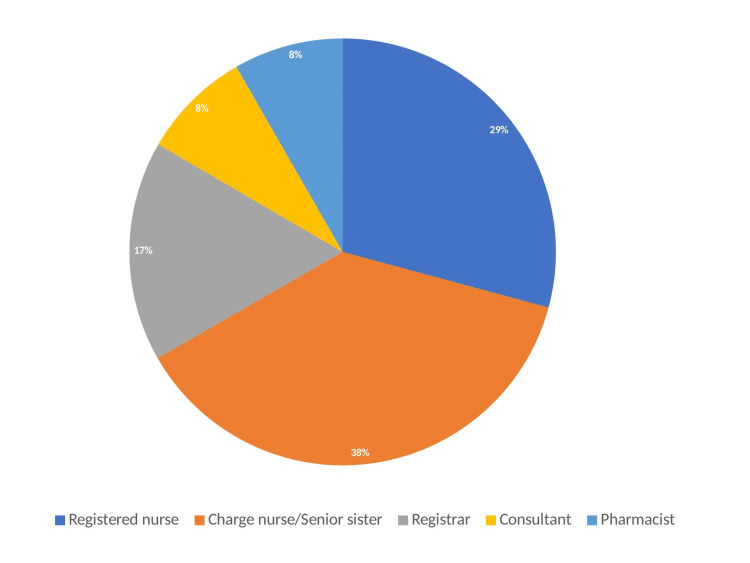
Pie chart detailing the staff roles of responders within our survey of all burns centres and units across the UK.

The majority of respondents were lead nurses or senior nurses (sisters) working in the burns ward (9/24) 37.5% within their department. Registered nurses working on the burns service accounted for 29.2% (7/24). 8.3% (2/24) of respondents were pharmacists. The remaining respondents were doctors within the department. 16.7% were specialty registrars in plastics and burns surgery (4/24) and the remaining 8.3% were burns or ITU consultants (2/24). The distribution of each respondent's role is shown in Figure 3. 

**Figure 4:**
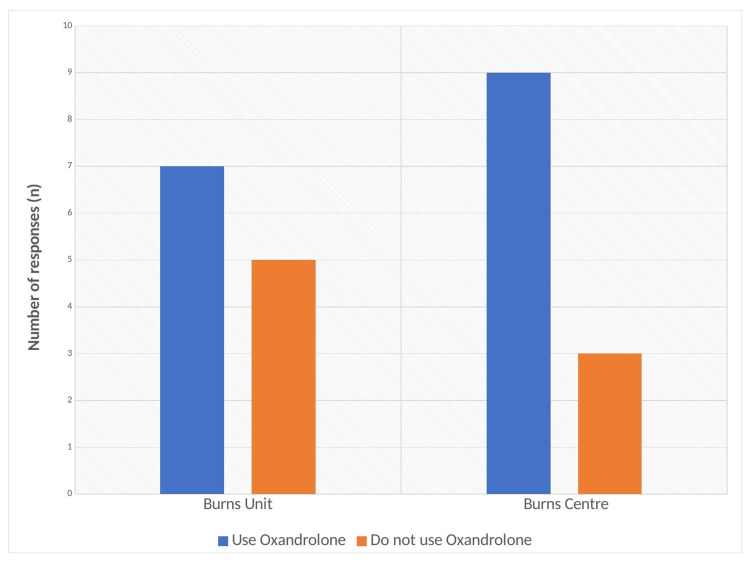
Graph detailing the number of responses depending on the type of burns service and whether they use Oxandrolone.

In total 16/24 (66.6%) services reported using Oxandrolone in the management of burns within their trust. Conversely, 8/24 (33.3%) burns services deny using Oxandrolone within their trust. This is sub-divided depending on the level of burns service provision and is displayed in Figure 4. Seven out of 12 (58.3%) burns units use Oxandrolone in the management of burns. On the other hand, five out of 12 (42.7%) burns units do not use Oxandrolone in severe burns. Within burns centres, 9/12 (75%) hospitals responded by using Oxandrolone, whilst 3/12 (25%) did not. 

**Figure 5:**
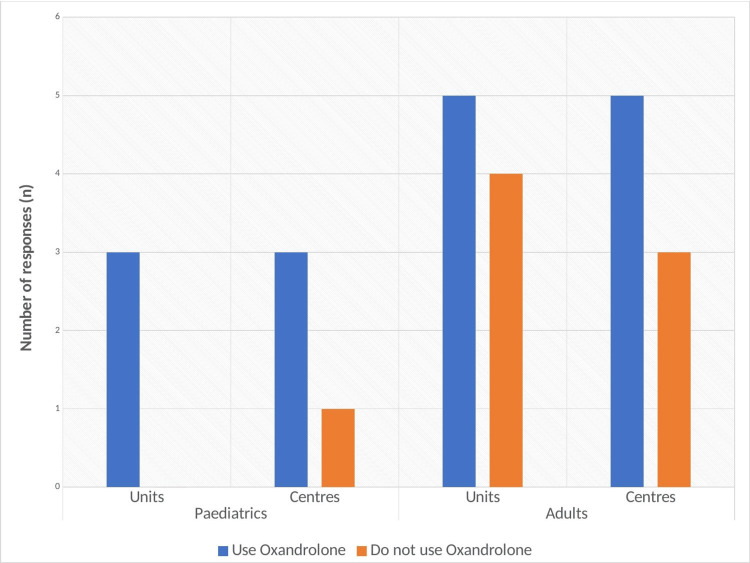
Column chart showing the use of Oxandrolone depending on the level of facility and subset of patients.

**Figure 6:**
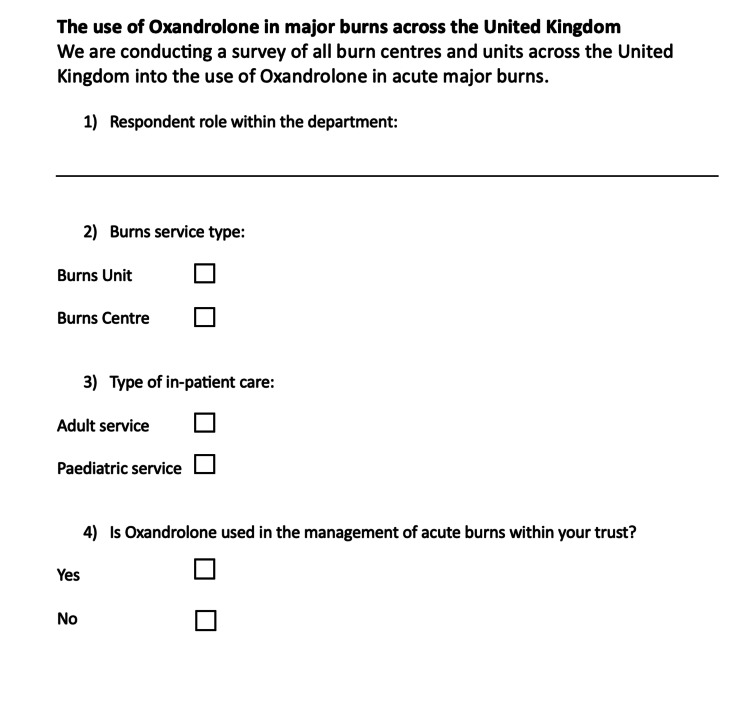
Questionnaire filled out by the authors of the study to collate information about Oxandrolone use across burns services within the UK.

The responses depending on the level of facility and use between adults or paediatric patients are shown in Figure 5. 3/4 (75%) paediatric burn centres surveyed use Oxandrolone, whilst 1/4 (25%) centres declare that they do not use Oxandrolone. All three paediatric units surveyed use Oxandrolone. In adult care, 5/8 (63%) adult burn centres responded that they do use Oxandrolone in the care of their patients. 3/8 (38%) of adult burn centres surveyed declared that they do not use Oxandrolone. 5/9 (56%) adult burn units surveyed use Oxandrolone, whilst 4/9 (44%) adult burn units do not use Oxandrolone. 

## Discussion

Our study indicates that Oxandrolone is used across the country in the acute management of severe thermal burns. In total, 66.6% of burn units and centres acknowledge the use of Oxandrolone in the management of their burns patients. Whilst 33.3% of units and centres deny the use of Oxandrolone within their practice. Our study shows that, despite not being licenced for use within the UK for acute burns, Oxandrolone is still prevalent across burns centres and units within the UK. 

Burn centres were more likely to use Oxandrolone in comparison to burn units. 75% of centres use Oxandrolone, whilst 25% do not. This was lower when burn units were surveyed, with 55% of respondent acknowledging the use of Oxandrolone within their practice and 45% not. This likely reflects the nature of the service. Burns centres manage, on a more frequent basis, patients with major resuscitation burns. These patients are induced into a profoundly hypercatabolic pathophysiological state. Burns centres are, therefore, presented with patients who would be amenable to Oxandrolone on a more frequent basis. 

Large burns are devastating injuries, which fortunately are not common. The decision to prescribe Oxandrolone requires senior specialist input as is usually made on a case-by-case basis. With this in mind, participant staff role was an important factor to take into consideration in our study. Oxandrolone is a daily prescription. Ward nurses who administer the drug, senior doctors and pharmacists should be familiar with patients receiving this medication. 41.6% of respondents were senior or charge nurses on the ward. Similarly, 29.2% were registered nurses who were familiar with the administration of this twice-daily drug. 16.7% and 8.3% of respondents were registrars and consultants, respectively; therefore, they should know whether Oxandrolone is prescribed in the trust, as they are usually involved in or aware of the reason for prescribing it. Lastly, 4.8% of respondents were pharmacists, who again should be aware of ordering stock and the provision of this medication that is prescribed daily.

Use varies across paediatric and adult services. 3/3 (100%) of paediatric burn centres described using Oxandrolone, with only 5/9 (55%) of adult burn centres using Oxandrolone. 3/4 (75%) of paediatric burn units describe using Oxandrolone, whilst 5/8 (63%) of adult units describe using Oxandrolone. Paediatric services are, therefore, more likely to use Oxandrolone within the UK. In spite of this, studies have suggested that the benefit of Oxandrolone is not age-dependent [13]. 

Many studies suggest that Oxandrolone is an effective adjuvant drug in the management of severe burns. It has a safe side effect profile and can be used in all age ranges, even at the extreme ends of age [14]. It has been shown to reduce mortality and significantly improve wound healing. This is a crucial property in severe burns and is associated with an increased risk of infection and patient morbidity [15]. In spite of this, Oxandrolone use across the UK appears to be extremely varied. Oxandrolone is not currently licenced for acute burns within the UK, and there are no national guidelines for its use. Despite this, the majority of burns centres and units across the UK use Oxandrolone. Further studies are needed to assess the efficacy of Oxandrolone and also how it is used in each trust, to assess whether there is a future for more generalised use of Oxandrolone for burns within the UK. 

Limitations

We encountered some difficulties with our survey. Firstly, there was the logistical difficulty of carrying out a national survey. We attempted to telephone clinical staff on wards at the hospitals and trusts outlined. This was difficult, due to the busy nature of the wards, meaning it was difficult to get a relevant member of staff to give up time to answer our questions. We set a limit of three attempts to reach a relevant healthcare professional. 

Another difficulty was locating specific guidelines on Oxandrolone use within specific trusts. Often, it was as if the trust did not have these guidelines given the specialised, case-by-case decision-making process. This meant we were unable to categorise how each trust was using Oxandrolone.

## Conclusions

In conclusion, the use of Oxandrolone varies greatly across services within the UK. Burns centres were more likely to use Oxandrolone in comparison to burns units. Further work is required to quantify the socioeconomic impact of Oxandrolone in the on-going management of patients with severe burns. National guidelines would further facilitate trusts and clinicians to use Oxandrolone appropriately and evert the disparity in its use across services within the UK.

## Appendices

Appendix 1

## References

[1] McCann C, Watson A, Barnes D (2022). Major burns: part 1. Epidemiology, pathophysiology and initial management. BJA Educ.

[2] Jeschke MG, Chinkes DL, Finnerty CC (2008). Pathophysiologic response to severe burn injury. Ann Surg.

[3] Nielson CB, Duethman NC, Howard JM, Moncure M, Wood JG (2017). Burns: pathophysiology of systemic complications and current management. J Burn Care Res.

[4] Jeschke MG, van Baar ME, Choudhry MA, Chung KK, Gibran NS, Logsetty S (2020). Burn injury. Nat Rev Dis Primers.

[5] Williams FN, Herndon DN, Jeschke MG (2009). The hypermetabolic response to burn injury and interventions to modify this response. Clin Plast Surg.

[6] Knuth CM, Auger C, Jeschke MG (2021). Burn-induced hypermetabolism and skeletal muscle dysfunction. Am J Physiol-Cell Physiol.

[7] Orr R, Fiatarone Singh M (2004). The anabolic androgenic steroid oxandrolone in the treatment of wasting and catabolic disorders: review of efficacy and safety. Drugs.

[8] Hart DW, Wolf SE, Ramzy PI (2001). Anabolic effects of oxandrolone after severe burn. Ann Surg.

[9] Wolf SE, Edelman LS, Kemalyan N (2006). Effects of oxandrolone on outcome measures in the severely burned: a multicenter prospective randomized double-blind trial. J Burn Care Res.

[10] Ring J, Heinelt M, Sharma S, Letourneau S, Jeschke MG (2020). Oxandrolone in the treatment of burn injuries: a systematic review and meta-analysis. J Burn Care Res.

[11] Pham TN, Klein MB, Gibran NS (2008). Impact of oxandrolone treatment on acute outcomes after severe burn injury. J Burn Care Res.

[12] British Burns Association (2023). UK Burns Services: Wakefield. https://www.britishburnassociation.org/Wakefield.

[13] Li H, Guo Y, Yang Z, Roy M, Guo Q (2016). The efficacy and safety of oxandrolone treatment for patients with severe burns: a systematic review and meta-analysis. Burns.

[14] Kopel J, Sorensen G, Griswold J (2022). A reappraisal of oxandrolone in burn management. J Pharm Technol.

[15] Ahmad A, Herndon DN, Szabo C (2019). Oxandrolone protects against the development of multiorgan failure, modulates the systemic inflammatory response and promotes wound healing during burn injury. Burns.

